# O-GlcNAcylation and O-GlcNAc Cycling Regulate Gene Transcription: Emerging Roles in Cancer

**DOI:** 10.3390/cancers13071666

**Published:** 2021-04-01

**Authors:** Matthew P. Parker, Kenneth R. Peterson, Chad Slawson

**Affiliations:** 1 Department of Biochemistry and Molecular Biology, University of Kansas Medical Center, Kansas City, KS 66160, USA; mparker6@kumc.edu; 2 Department of Anatomy and Cell Biology, University of Kansas Medical Center, Kansas City, KS 66160, USA; 3 KU Cancer Center, University of Kansas Medical Center, Kansas City, KS 66160, USA

**Keywords:** transcription, O-GlcNAc, O-GlcNAc transferase, O-GlcNAcase

## Abstract

**Simple Summary:**

O-linked β-N-acetylglucosamine (O-GlcNAc) is a post-translational modification (PTM) linking nutrient flux through the hexosamine biosynthetic pathway (HBP) to gene transcription. Mounting experimental and clinical data implicates aberrant O-GlcNAcylation in the development and progression of cancer. Herein, we discuss how alteration of O-GlcNAc-regulated transcriptional mechanisms leads to atypical gene expression in cancer. We discuss the challenges associated with studying O-GlcNAc function and present several new approaches for studies of O-GlcNAc-regulated transcription.

**Abstract:**

O-linked β-N-acetylglucosamine (O-GlcNAc) is a single sugar post-translational modification (PTM) of intracellular proteins linking nutrient flux through the Hexosamine Biosynthetic Pathway (HBP) to the control of cis-regulatory elements in the genome. Aberrant O-GlcNAcylation is associated with the development, progression, and alterations in gene expression in cancer. O-GlcNAc cycling is defined as the addition and subsequent removal of the modification by O-GlcNAc Transferase (OGT) and O-GlcNAcase (OGA) provides a novel method for cells to regulate various aspects of gene expression, including RNA polymerase function, epigenetic dynamics, and transcription factor activity. We will focus on the complex relationship between phosphorylation and O-GlcNAcylation in the regulation of the RNA Polymerase II (RNAP II) pre-initiation complex and the regulation of the carboxyl-terminal domain of RNAP II via the synchronous actions of OGT, OGA, and kinases. Additionally, we discuss how O-GlcNAcylation of TATA-box binding protein (TBP) alters cellular metabolism. Next, in a non-exhaustive manner, we will discuss the current literature on how O-GlcNAcylation drives gene transcription in cancer through changes in transcription factor or chromatin remodeling complex functions. We conclude with a discussion of the challenges associated with studying O-GlcNAcylation and present several new approaches for studying O-GlcNAc regulated transcription that will advance our understanding of the role of O-GlcNAc in cancer.

## 1. O-GlcNAcylation Is a Post-Translational Modification (PTM) That Has Regulatory Roles in Gene Transcription

Cells precisely control gene expression in response to their metabolic state, the availability of building blocks and fuel, and environmental cues [1]. Eukaryotic gene transcription is controlled by many proteins, including the basal transcription machinery, epigenetic chromatin remodeling complexes, and transcription cofactors [2]. O-linked β-N-acetylglucosamine (O-GlcNAc) has been found on proteins in all these groups and is involved with virtually every step of transcription [1]. O-GlcNAc is a PTM in which a single O-GlcNAc moiety is attached to serine and threonine residues of cytoplasmic, nuclear, and mitochondrial proteins [3,4,5]. Much progress has been made in our understanding of the biochemical, molecular, and physiological effects of O-GlcNAcylation on gene transcription over the past decade due, in part, to the development of improved genetic and pharmacological tools to study its function, which has rapidly accelerated interest in this PTM and its involvement in gene regulation.

O-GlcNAc cycling describes the rapid addition and removal of the sugar from intracellular proteins, which affects their interactions, stability, localization, and activity. O-GlcNAc is covalently attached to substrate proteins by a single enzyme, *O*-linked *N*-acetylglucosamine transferase (OGT; also known as O-GlcNAc transferase), and removed by a single enzyme, *N*-acetyl-β-glucosaminidase (OGA; also known as O-GlcNAase) [6]. There are, however, different isoforms of OGT and OGA, the functional significance of which are beyond the scope of this review. Both enzymes are indispensable for cellular growth, development, and in most cases, survival [5,7,8,9]. In rapidly dividing tissue, the loss of OGT or OGA is lethal, making both enzymes attractive cancer therapy targets [7].

PTMs are often regulated and/or exert their effect through an assortment of proteins known as “writers”, “readers”, and “erasers”. Simplistically, “writers” add the PTM to substate proteins, whereas “erasers” remove the modification. For example, kinases add phosphate groups to target proteins, and phosphatases remove the phosphate group from proteins. “Readers” recognize the PTM via “reader domains” and integrate the molecular and chemical information of the PTM to drive cellular processes by recruiting effector proteins and enzymes. For example, proteins with SRC homology 2 (SH2) domains recognize and bind phosphate PTMs [10]. These “writer”, “reader”, and “eraser” functions can exist in a single protein or in multi-protein complexes to control essential cellular processes. The addition and removal of O-GlcNAc marks, which are found on thousands of proteins, is accomplished by a single pair of enzymes, one “writer” and one “eraser”. Until recently, there has been little evidence for proteins with an O-GlcNAc “reader domain” to facilitate interaction with O-GlcNAcylated proteins. Toleman et al. identified several human protein candidates, including 14-3-3 isoforms, that bind O-GlcNAc directly and selectively [11]. Despite nearly four decades of work in the field, outside this example, little experimental evidence exists for O-GlcNAc “reader domains”. Thus, the existence of O-GlcNAc “reader domains” remains controversial and requires further exploration.

In the absence of O-GlcNAc “reader” proteins, other possibilities regarding the function of protein O-GlcNAcylation must be considered. There is considerable evidence to support the notion that O-GlcNAcylation may sterically promote or prevent protein-protein interactions. This mechanism is suggested by the wide range of biochemical changes O-GlcNAcylation induces in its protein targets, including inhibition or activation of activity, conformational changes, re-localization, and destruction [5,8,9,12,13,14]. Evidence also indicates that O-GlcNAcylation engages in a complex interplay with other PTMs, most notably, phosphorylation, to potentiate and govern a diverse array of transcriptional and cellular processes. O-GlcNAcylation is almost as common as phosphorylation and is often found in a reciprocal relationship with phosphorylation on the same serine/threonine residues [15]. Experimental data indicate that the interplay between PTMs or the “PTM code” extends beyond the level of single proteins and may coordinate protein complex formation, function, and activity [16]. Thus, the question remains, how does O-GlcNAc manifest itself functionally in gene transcription, and in what way are the transcriptional functions of O-GlcNAc dysregulated in cancer?

## 2. O-GlcNAcylation Is Sensitive to Metabolite Pools via HBP

**Figure 1:**
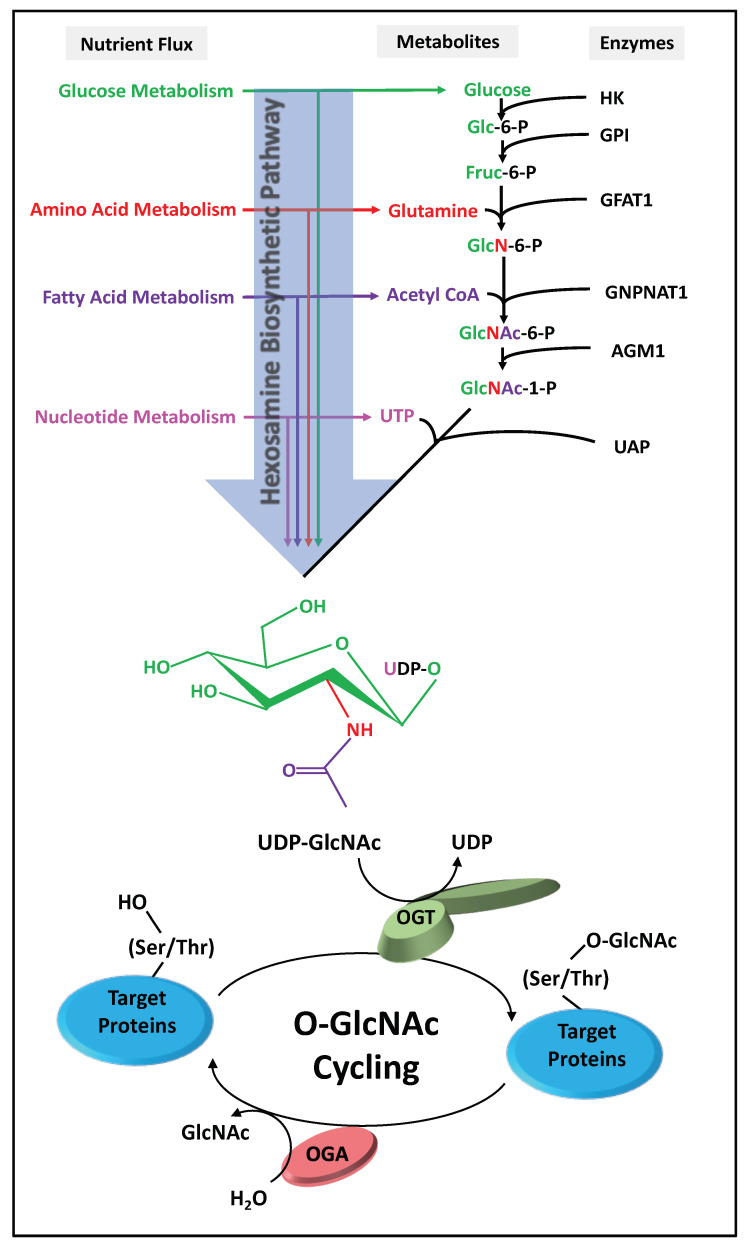
The Hexosamine Biosynthetic Pathway (HBP) and O-GlcNAc Cycling. Glucose enters the cells and is enzymatically converted to Glucose-6-phosphate (Glc-6-P) by Hexokinase (HK). Glucose-6-phosphate isomerase (GPI) then converts Glc-6-P to Fructose-6-phosphate (Fruc-6-P), after which approximately 95% of it proceeds to glycolysis and 3–5% is converted to Glucosamine-6-phosphate (GlcN-6-P) by the enzyme Glutamine fructose-6-phosphate amidotransferase (GFAT). Glutamine is required for this enzymatic reaction. This enzymatic reaction also constitutes the rate-limiting step of the HBP. Glucosamine-6-phosphate *N*-acetyltransferase 1 (GNPNAT1) then utilizes acetyl-CoA to convert GlcN-6-P into *N*-acetylglucosamine-6-phosphate (GlcNAc-6-P). This is then converted to *N*-acetylglucosamine-1-phosphate (GlcNAc-1-P) by Phosphoacetylglucosamine mutase 1 (AGM1). Uridine triphosphate (UTP) is then utilized by UDP-*N*-acetylglucosamine pyrophosphorylase (UAP) to convert GlcNAc-1-P to Uridine diphosphate *N*-acetylglucosamine (UDP-GlcNAc). O-GlcNAc transferase (OGT) and O-GlcNAcase (OGA) facilitate O-GlcNAc cycling “on and off” serine and threonine amino acid residues of target proteins.

Changes in intracellular nutrient metabolite pools directly affect the level uridine-diphosphate *N*-acetyl glucosamine (UDP-GlcNAc), the substrate for OGT and other glycosyltransferases, produced by the HBP [17,18]. The HBP utilizes products from amino acid, fatty acid, nucleotide, and glucose metabolism to generate UDP-GlcNAc. Since multiple metabolites feed into the HBP, UDP-GlcNAc levels are responsive to fluctuations in these metabolic pathways (Figure 1) [19,20,21]. For example, increasing glucose availability induces a rapid increase in intracellular UDP-GlcNAc levels in a variety of cell types [22,23,24,25]. In contrast, glucose depletion results in a reduction of UDP-GlcNAc levels [17]. Similar to glucose, changes in the intracellular glutamine, fatty acid, and nucleotide pools also lead to a modulation of HBP flux and UDP-GlcNAc levels [17]. The prevailing hypothesis in the O-GlcNAc field is that UDP-GlcNAc levels act as a proxy for overall nutrient availability in the cells. Consequently, O-GlcNAcylation can exert pressure on many cellular processes, including gene regulatory mechanisms, in response to changing nutrient and metabolic demands. Interestingly, dysregulated cellular energetics and altered metabolism are now considered a hallmark of all cancers [26]. Every cancer type studied to date has aberrant O-GlcNAc cycling, and a growing number of studies suggest that O-GlcNAcylation constitutes an important regulator of cancer growth and progression [1,27,28]. However, the clinical importance of shifts in cellular O-GlcNAc levels and how these levels are involved with the onset, progression, and metastasis of cancer are still largely unknown [29].

## 3. O-GlcNAcylation Regulates RNA Polymerase Function

At the basal level of transcription, eukaryotic gene expression is governed by three different evolutionarily conserved RNA polymerases (RNAPs). Each RNAP transcribes different types of genes in the genome. RNA polymerase I (RNAP I) transcribes ribosomal RNA genes (rRNAs). RNA polymerase II (RNAP II) transcribes a variety of DNA elements, including messenger RNAs (mRNAs), microRNAs (miRNAs), small nuclear RNAs (snRNAs), and small nucleolar RNAs (snoRNAs). RNA polymerase III (RNAP III) produces transfer RNAs (tRNAs) and 5S ribosomal RNAs (5S rRNAs) [30]. Each RNAP has a dedicated set of core transcription factors that recognize specific conserved promoter elements. These transcription factors and core promoter elements are required to form the pre-initiation complex (PIC) and initiate RNA synthesis.

### 3.1. O-GlcNAcylation and RNAP II Function

Of all the RNAPs and their associated synthesis machinery, eukaryotic RNAP II is the most versatile, as it recognizes and catalyzes transcription from the most diverse set of gene promoters [31]. It also transcribes the widest dynamic range of mRNA expression levels, ranging from just a few mRNA copies for some genes to millions of copies for others [32]. Control of RNAP II activity occurs at many different levels, including recruitment to promoters, PIC formation, transcription initiation, elongation, splicing, and termination. The way in which RNAP II progressively movies through these events is referred to as the transcription cycle. PTMs on the carboxyl-terminal domain (CTD) of RNAP II play a major role in the orchestration of these events. In humans, the CTD of RNAP II consists of 52 imperfect heptad amino acid repeats of predominantly YSPTSPS [33,34,35]. The phosphorylation status of the CTD defines different forms of RNAP II. RNAP IIA is the unphosphorylated form, and RNAP IIO is the phosphorylated form. Early RNAP II transcriptional cycling models established in the late 1980s and early 1990s suggested that RNAP IIA was the RNAP II spices recruited to gene promoters and required for PIC formation, whereas RNAP IIO was generated later in the transcription cycle during transcriptional initiation [36]. In addition to RNAP IIA and RNAP IIO, a third O-GlcNAcylated species of RNAP II (RNAP IIγ) was identified by Kelly et al. [37]. However, the role of this RNAP species has remained unknown until recently.

**Figure 2:**
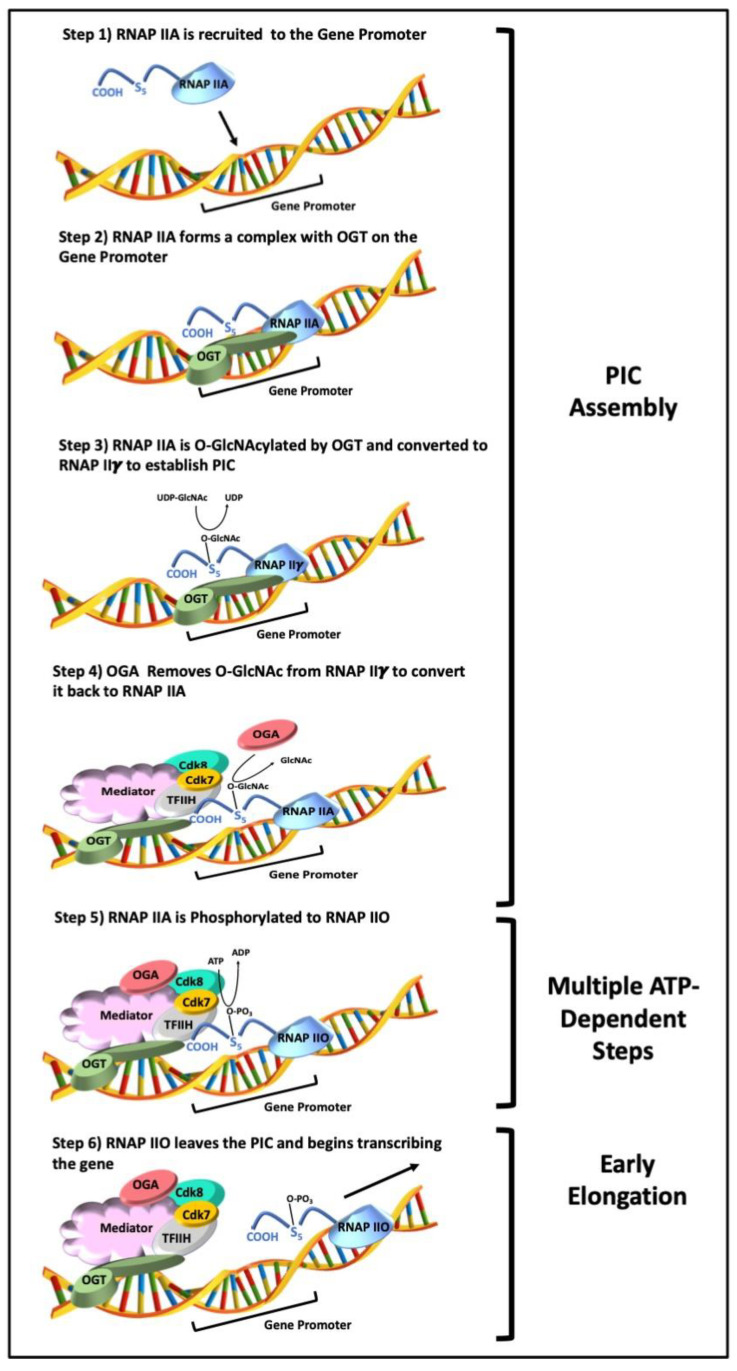
O-GlcNAc cycling and RNAP II-mediated transcription. Model demonstrating how O-GlcNAc cycling regulates various steps of RNAP II transcriptional initiation. In this model, RNAP IIA (unmodified) is recruited to gene promoters by basal transcription factors and core promoter elements (Step 1). RNAP IIA then interacts with OGT (Step 2) and is then O-GlcNAcylated, generating RNAP IIγ (Step 3). OGT may be part of the basal transcription factors at the core promoter elements or is recruited to these *cis*-regulatory elements after RNAP IIA is bound. O-GlcNAcylation of RNAP II, and possibly other basal transcription proteins, lead to the formation of the PIC. OGA associates with the PIC to convert RNAP IIγ back to RNAP IIA (Step 4). Finally, RNAP IIA is phosphorylated to generate RNAP IIO (Step 5). RNAP IIO is released from the PIC to initiate transcription (Step 6).

Using in vitro cell-free systems derived from crude HeLa cells extracts, Lewis et al. explored the function of RNAP IIγ in the transcription cycle, resulting in a revision of the early model to include O-GlcNAc cycling [15,33,38,39]. In this model, RNAP IIA associates with DNA promoters early in the transcription cycle. RNAP IIA is then O-GlcNAcylated to generate RNAP IIγ during PIC formation. RNAP IIγ is then converted back to the non-O-GlcNAcylated form RNAP IIA by OGA before transcriptional initiation (Figure 2). Both OGT and OGA enzymes activity is essential for PIC formation since inhibition of O-GlcNAc cycling by OGT and OGA inhibitors resulted in transcription inhibition [15,38]. In addition, Lewis et al. found that OGA physically interacts with the elongation factors SPT5 and TIF1β and maps to promoters genome-wide [39]. Human ChIP-seq genomic consortia have shown that O-GlcNAc, OGA, and OGT peaks clearly overlap with the 5′ end of human genes and co-localize with RNAP II peaks. The co-localization of O-GlcNAc, OGA, OGT, and RNAP II at the 5′ end of genes is also observed in *M. musculus*, *Drosophila*, and *C. elegans*, further demonstrating a role for O-GlcNAc cycling in gene transcription that seems to be evolutionally conserved in Eukarya [39,40].

Functionally, O-GlcNAcylation of the RNAP II CTD may regulate PIC assembly, transcriptional pausing, and elongation in several possible ways. First, O-GlcNAcylation prevents phosphorylation by impeding access of kinases to the necessary serine and threonine residues of the CTD. Phosphorylation of these amino acid residues are required for RNAP II to dissociate from the PIC and critical for mRNA processing and transcription cycle progression. Second, O-GlcNAc addition and removal may promote or impede protein-protein interactions required for PIC assembly, transcriptional pausing, and elongation. Thus, this mechanism may be operative in cells to control which genes are turned off and on, or which gene splice variants are expressed under different internal cellular and external environmental conditions. Beyond RNAP II, mass spectroscopy data have identified 32 additional RNAP II transcription cycling factors that are O-GlcNAcylated [41]. However, little is known about how these modifications affect various aspects of the transcription cycle. Together, these experiments support the notion that there may be a direct connection between the cellular nutrient state, RNAP II function, and transcriptional regulation. In this paradigm of nutrient-regulated gene transcription, the RNAP II transcriptional machinery is constantly sampling the cellular environment via O-GlcNAcylation to govern various aspects of PIC formation, transcription initiation, elongation, splicing, and termination. Based on the data thus far, it is unclear which promoters utilize a GlcNAc-dependent transcription cycling step. To date, only a small number of promoters in cell-free systems have been extensively analyzed. In light of this data, another layer of plasticity must be considered when thinking about the basal transcriptional machinery and how cells might incorporate different environmental and metabolic cues to control gene transcription. In transformed metabolic states such as the Warburg effect and in the microenvironment of solid tumors, these RNAP II nutrient-sensing mechanisms would undoubtedly behave abnormally, leading to altered gene expression and could promote the progression and metastasis of cancer. O-GlcNAcylation may be a mechanism cancer cells use to manipulate their metabolism to promote survival, proliferation, and long-term maintenance in various environmental conditions.

### 3.2. O-GlcNAcylation of TATA-Box Binding Protein (TBP) Alters Metabolic Gene Expression

**Figure 3:**
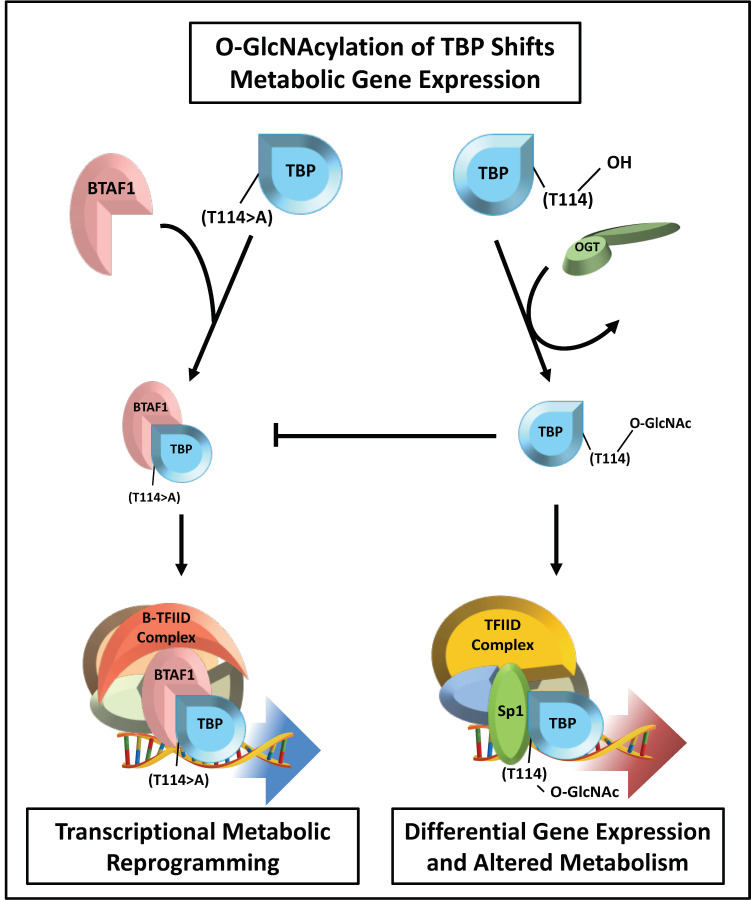
O-GlcNAcylation of TBP regulates metabolic gene expression. O-GlcNAcylation of TBP at the amino acid residue T114 impairs B-TFIID complex formation by disrupting BTAF1 binding. Disruption of TBP O-GlcNAcylation results in significant metabolic transcriptome reprogramming, leading to a profound alteration in lipid storage. Using O-GlcNAc as a nutrient sensor, cells can fine-tune the metabolic transcriptome. Dysregulation of O-GlcNAc signaling has catastrophic effects on metabolic gene expression.

A protein factor common to all three RNAP complexes is the TBP, which is thought to serve as a PIC assembly platform in TATA-box containing promoters. TBP associates with two RNAP II transcriptional complexes, TFIID and B-TFIID [42,43]. Each complex has unique TBP-associated factors that aid in promoter selectivity. TFIID and B-TFIID are not equivalent; TFIID is responsive to gene-specific transcription factors such as SP1, while B-TFIID is not. Recently, Hardiville et al. identified an O-GlcNAc site in the N-terminal domain (NTD) of TBP at T114 [43]. O-GlcNAcylation at this amino acid residue blocks the ability of TBP to interact with TATA-box binding protein associated factor 1 (BTAF1), which disrupts B-TFIID complex formation. To test the functional significance of O-GlcNAcylation at this amino acid residue, cellular O-GlcNAcylation was increased by inhibiting OGA activity with Thiamet G (TMG). Increasing TBP O-GlcNAcylation had no effect on nuclear localization; however, there was a substantial increase in DNA binding and a significant reduction in BTAF1 interaction. To reduce TBP O-GlcNAcylation, OGT was inhibited with Ac4SGlcNAc, which resulted in decreased TBP chromatin immobilization and increased BTAF1 binding. This data suggests that O-GlcNAc can regulate RNAPs by modulating TBP DNA binding and B-TFIID complex formation. CRISPR/Cas9 mutagenesis of the T114A O-GlcNAc site on TBP increased TBP binding to BTAF1 and directly impacted the expression of 408 genes. This mutation led to profound reprogramming of cellular metabolism and alterations in lipid storage (Figure 3) [43]. This study reveals that the NTD of TBP can integrate nutrient signals via O-GlcNAcylation, to modulate cell metabolism by adjusting gene expression programs.

Phosphorylation and O-GlcNAcylation have extensive crosstalk with each other [5,44,45,46]. Experimental evidence suggests that the simultaneous phosphorylation of the NTD of TBP and transcription factor II B (TFIIB), a general transcription factor involved in the formation of PICs, might stimulate transcription initiation of certain RNAP II regulated genes [47]. However, phosphorylation of TBP alone may result in transcriptional silencing of RNAP II regulated genes during mitosis [48]. In this regard, PTM marks may constitute a complex “code” on the NTD of TBP that fine-tune its activity and promote or obstruct interaction of TBP with specific proteins or protein complexes, thereby controlling the expression of genes regulated by these interactions [43]. In the context of nutrient-regulated gene transcription, this data provides additional evidence of a connection between cell metabolism and transcriptional regulation. Under atypical cellular O-GlcNAc conditions, such as those commonly observed in cancer, the activity of TBP, TFIIB, and genes sensitive to the phosphorylation and O-GlcNAc states of these proteins are at risk for anomalous expression. The metabolic reprogramming mechanism presented by Hardiville et al. [43] may be involved with cancer pathology, but the question remains whether aberrant O-GlcNAcylation is a driver or a consequence of upstream events that led to the manifestation of cancer.

## 4. O-GlcNAcylation and Epigenetic Gene Regulators

Cancer results from a combination of changes to the genome and epigenome [49]. Epigenetics is defined as the study of changes in gene expression due to mechanisms other than DNA sequence mutations. The alterations in gene expression associated with epigenetics are governed by changes in chromatin structure. Chromatin consists of DNA and its associated proteins, most notably, histones. Epigenetic “marks” are the complete set of DNA and histone modifications that modulate the affinity of chromatin-binding proteins by altering chromatin structure [50,51,52]. Nutrient flux affects gene expression through epigenetic mechanisms [53]. For example, histone acetyltransferases (HATs) transfer the acetyl group from acetyl-CoA to the lysine residues of histones, thereby neutralizing the positive charge on lysine residues. This reduces the ionic interaction between histones and the DNA sugar-phosphate backbone, which leads to a more relaxed open chromatin conformation. Acetyl-CoA is a metabolite produced by nutrient flux through carbohydrate and lipid metabolic pathways. Elevated levels of acetyl-CoA stimulate cellular growth by prompting histone acetylation of genes controlling growth, which increases the expression of these genes. Chromatin and epigenetic regulatory complexes have been identified as O-GlcNAc targets [54]. O-GlcNAc is also part of the so-called “histone code”. Several O-GlcNAc sites have been mapped to histone tails and the histone-DNA interface [53]. Evaluation of histone proteins in HeLa cell nuclear extracts identified O-GlcNAc on histone H2A, H2B, H3, and H4 [55]. Perturbing O-GlcNAc cycling by manipulating OGT and OGA expression leads to alteration of various histone modifications, such as H3K9 acetylation, H3S10 phosphorylation, and H3R17/K27 methylation, strongly implicating O-GlcNAc in epigenetic gene regulatory mechanisms [55,56].

### 4.1. O-GlcNAcylation of Ten-Eleven Translocation Protein Family

Methylation of cytosines in DNA, particularly at promoter CpG islands, is the classical epigenetic modification that plays a critical role in transcription, affecting such downstream processes as chromosome accessibility, nucleosome positioning, and ultimately, gene expression [57]. In humans, three members of the ten-eleven translocation protein family (TET1/2/3) catalyze the sequential oxidation of 5-methylcytosine to 5-hydroxymethylcytosine, 5-formylcytosine, and 5-carboxylacytosine, which serve an essential role in embryonic development and tumor progression [58]. TET proteins interact with OGT and undergo O-GlcNAcylation, which alters their activity and stability [59]. In addition, TET proteins form complexes with OGT and shuttle OGT to specific loci where target proteins are O-GlcNAcylated. For example, Chen et al. have shown that TET2 mediates O-GlcNAcylation of histone H2B at S112 at highly transcribed genes [59]. Deplus et al. showed a direct physical interaction between OGT and TET2/3 proteins and proposed a hierarchical model for H3K4me3 and transcriptional activation [60]. Initially, a TET2 or TET3-OGT complex is formed, which is targeted to DNA loci by an unknown mechanism. OGT does not appear to influence TET2/3 enzymatic activity or targeting; rather, TET2/3 proteins target and promote O-GlcNAcylation of numerous proteins, including host cell factor 1 (HCF1). HCF1 is essential for the recruitment of the SET1/COMPASS complex. O-GlcNAcylation of HCF1 stabilizes the SET1/COMPASS complex and promotes H3K4me3 and subsequent transcriptional activation. Perturbation of TET2/3 or OGT activity results in a direct decrease in H3K4me3 and a concomitant decrease in transcription [60]. More recently, Bauer et al. mapped O-GlcNAc sites on all of the TET proteins [61]. Besides O-GlcNAcylation, TET proteins also were found to be highly phosphorylated, with each TET protein having its own unique phosphorylation pattern or “code” [61]. As O-GlcNAc and phosphorylation are mutually exclusive marks, OGA and, therefore, O-GlcNAc cycling may play an important role in regulating phosphorylation. Collectively, these data show that O-GlcNAcylation is intricately involved with TET1/2/3 protein functions; however, the precise mechanism by which the TET protein-OGT-OGA protein axis works is unclear. The H3K4me3 SET1/COMPASS mechanism proposed by Deplus et al. sheds new light on how TET, OGT, and epigenetic mechanisms work together to regulate gene expression. Thus, dysregulation of O-GlcNAc in cancer would directly affect the function of TET proteins and their target genes [60].

### 4.2. O-GlcNAcylation of Polycomb Group Proteins

The polycomb group (PcG) proteins are a diverse family of epigenetic modifiers and transcriptional regulators. In mammals, PcG proteins repress Hox genes and control the expression of other genes that govern embryonic development, X chromosome inactivation, genomic imprinting, the cell cycle, and maintenance of stem cells [62]. Different PcG protein complexes contain distinct chromatin-modifying activities that contribute to the formation of repressive heterochromatin. Dysregulation of PcG proteins plays a role in oncogenesis and is associated with poor patient prognosis [63,64]. Evidence for interaction of OGT with PcG complexes was first observed in Drosophila, where deletion of Ogt led to body plan defects, specifically, abnormal patterning of the anteroposterior axis, a phenotype commonly associated with polycomb gene defects [65]. ChIP-seq experiments performed on Drosophila larvae identified 490 polycomb response elements (PREs) where PcG proteins and OGT co-localized. This data suggested that O-GlcNAcylation is involved with PcG-mediated repression of Hox genes [66,67,68]. Recently, Geo et al. mapped the OGT interactome in Hela cells and found that OGT interacts with numerous PcG proteins, including those belonging to PRC1, PRC2, PR-DUB, and PhoRC complexes [69]. In an unrelated study, Hauri et al. identified two human PRC2 complexes and two PR-DUB deubiquitylation complexes that contained OGT [70]. Additionally, Forma et al. suggested that EZH2, the catalytic component of the PRC2 complex, interacts with OGT in a cell type-specific manner to regulate a subset of PRC2 target genes such as FOXA1 and FOXC1, two genes that are often dysregulated in hormone-dependent cancers [71]. Interestingly, Jiang et al. found that both OGT and EZH2 are post-transcriptionally inhibited by microRNA-101 (miR-101) [72]. Accumulation of O-GlcNAc, EZH2, and H3K27me3 in the miR-101 promoter region inhibits the transcription of miR-101 and result in upregulation of OGT and EZH2. In colorectal cancer (CRC), elevated protein levels of OGT and EZH2 have been shown to potentiate gene dysregulation and promote CRC metastasis [72]. Thus, manipulation of this regulatory circuit may be a potential therapeutic strategy for metastatic CRC [72]. Decourcelle et al. recently presented additional evidence for OGT and EZH2 involvement in CRC metastasis [73]. They found that O-GlcNAcylated EZH2 transcriptionally repressed UNC5A, a tumor suppressor gene frequently epigenetically downregulated in CRC. This study provides additional evidence for a link between nutrition and O-GlcNAc-mediated epigenetic regulatory mechanisms.

Among the OGT-interacting PcG proteins, several are direct targets of OGT and thus are sensitive to O-GlcNAcylation. Pei-Wen et al. have shown that EZH2 is O-GlcNAcylated in the N-terminal region at S73, S76, S84, and S87, which protects EZH2 from ubiquitin-proteasome degradation [74]. Additionally, Chi-Shuen et al. showed that O-GlcNAcylation of EZH2 at S75 is required for EZH2 protein stability and indirectly facilitates PRC2-mediated gene repression [75]. Moreover, O-GlcNAcylation in the catalytic domain at S729 was found to be essential for EZH2 methyltransferase activity, indicating that O-GlcNAc has a direct effect on EZH2 enzymatic function [74]. Lastly, Maury et al. highlighted the importance of O-GlcNAc on RING1B, which is the catalytic subunit of PRC1 complexes [76]. During human embryonic stem cell (hESC) differentiation, O-GlcNAcylation of RING1B decreases at T250/S251. ChIP-seq results show that non-O-GlcNAcylated RING1B is enriched near cell cycle genes, whereas O-GlcNAcylated RING1B co-localizes to neuronal genes [76]. This suggests that O-GlcNAcylation is a mechanism that targets PRC1 complexes to specific loci.

The role of O-GlcNAc cycling in PcG complex formation and function has recently been evaluated in Drosophila. Using RNAi to knockdown Ogt and Oga, Akan et al. reported that PcG-mediated repression was strikingly insensitive to Oga RNAi, in contrast to Ogt RNAi, which suggested that the addition of O-GlcNAc, and not the removal, is essential for PcG complex formation and function [67]. There are several hypotheses that may account for this observation. First, the O-GlcNAc moieties in PcG complexes may be inaccessible to OGA, and therefore, insensitive to Oga perturbation. Second, the level of RNAi knockdown is not sufficient to produce a phenotype in PcG complexes. Third, Oga and O-GlcNAc cycling is not required for PcG complex formation and function. The overall conclusion of all the data summarized in this section demonstrates that O-GlcNAcylation regulates PcG complexes at many different levels and that there is an intricate relationship between O-GlcNAc and PcG proteins. Further work is required to clarify the roles of O-GlcNAcylation in PcG-mediated gene regulation, especially during tumorigenesis and cancer progression.

### 4.3. O-GlcNAcylation of GATA1 Target Genes and the Sin3A Corepressor Complex

O-GlcNAc cycling exerts effects on gene transcription via protein-protein interaction with co-repressor and co-activator complexes and lineage-specific master transcription factors that function as adapter proteins to shuttle OGT and OGA to specific loci. An example of this is the Sin3A co-repressor complex, and more recently, erythroid-specific GATA-1 complexes [77,78]. Zhang et al. found that GATA-1, an essential master regulator of erythropoiesis, formed a complex with OGT and OGA when erythroid differentiation was induced [77]. When OGA function was disrupted with TMG, red blood cell maturation was impaired. Zhang et al. hypothesized that GATA-1 facilitated O-GlcNAc cycling at specific loci by delivering OGT and OGA to gene promoters and argued that cells might employ this gene expression regulatory mechanism to drive cell differentiation programs [77]. Little is known about this newly discovered mechanism, and as such, more research is needed to explore its relevance in cellular differentiation and its potential function in hematologic malignancies.

Sin3A is a core component of several transcriptional co-repressor complexes [79]. One of these complexes is the Sin3A-histone deacetylase (Sin3A/HDAC) containing complex. Sin3A physically interacts with, and is O-GlcNAcylated by, OGT [78,80]. How Sin3A/HDAC and OGT activities work to silence genes is not fully understood. O-GlcNAcylation and Sin3A/HDAC activity are thought to work synergistically to regulate transcription factor binding, activator/repressor complex formation and activity, and RNAP II function. Yang et al. has proposed a model for how this complex may regulate gene expression [78]. In this model, sequence-specific repressors bind to gene promoters and recruit the OGT containing Sin3A/HDAC complex. O-GlcNAcylation of these repressors could facilitate and stabilize protein-protein interactions critical for gene repression. Alternatively, or simultaneously, O-GlcNAcylation of activator complexes at gene promoters may disrupt essential hydrophobic interactions and trigger disassembly of these activator complexes, thereby increasing Sin3A/HDAC accessibility to histone targets. OGT activity at the promoter could also potentially arrest RNAP II activity by blocking phosphorylation sites, thereby preventing downstream transcription cycle events. Additionally, O-GlcNAcylation of Sin3A might regulate HDAC activity. Overall, the role of OGT in the Sin3A/HDAC complex could ultimately serve to couple nutrient signals to histone deacetylation activity, thereby regulating gene expression to ensure genes are silenced in an efficient, specific, and nutrient-regulated manner. The discovery of OGT containing Sin3A/HDAC complexes and the model proposed by Yang et al. [78], serve as an example of how O-GlcNAc might collaborate with epigenetic regulatory complexes in general. More experimental data is needed to refine our understanding of how OGT containing epigenetic complexes function, and further exploration is required to examine whether these regulatory mechanisms are dysregulated in metabolic diseases and cancer.

### 4.4. O-GlcNAcylation of Nucleosome Remodeling Deacetylase

**Figure 4:**
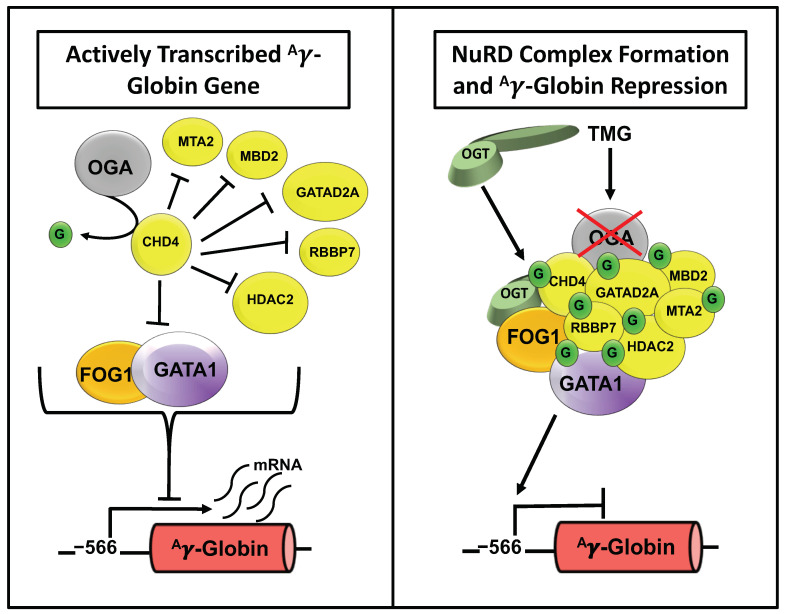
O-GlcNAcylation regulates the formation and function of the NuRD complex. ^A^γ-globin expression is silenced by the recruitment of NuRD complex to the –566 GATA binding site. OGA removes O-GlcNAc (green circle “G”) from CHD4 (a subunit of the NuRD complex), thereby preventing the assembly of the NuRD complex at the –566 site (left panel). When OGA is inhibited by TMG, OGT associates and O-GlcNAcylates CHD4, stimulating the formation of the NuRD complex at the –566 site, which represses ^A^γ-globin gene transcription (right panel). Additional O-GlcNAc sites have been found on every subunit of the NuRD complex, however, their function remains unknown.

The nucleosome remodeling deacetylase (NuRD) complex is a group of associated proteins with both ATP-dependent chromatin remodeling and histone deacetylase activities that relies on O-GlcNAcylation to regulate its function. NuRD was originally defined as a repressor, but more recently, it has been shown to function both as a co-activator and co-repressor [81]. The core protein constituents of NuRD consist of CHD3/4/5, CDK2AP1, GATAD2A/B, and MBD2/3, which bridges the remodeling subcomplex to the histone deacetylase subcomplex. The composition of the histone deacetylase subcomplex consists of HDAC1/2, MTA1/2/3, and RBBP4/7 proteins [82]. Recent studies have identified O-GlcNAc on every protein of the NuRD complex [69,83]. Additionally, Geo et al. [69] and Zhang et al. [83] demonstrated that OGT directly interacts with the NuRD complex. Zhang et al. showed that when K562 cells were treated with TMG, γ-globin gene expression was reduced, and NuRD occupancy at the promoter increased [83]. OGA was found to interact with NuRD under all conditions tested; however, OGT only interacted with NuRD when γ-globin was silenced [83]. They also demonstrated that O-GlcNAcylation of CHD4 stimulated NuRD complex formation and gene repression of the γ-globin locus, whereas removal of O-GlcNAc from CHD4 by OGA had an activating effect on γ-globin gene expression (Figure 4) [83]. This study provides evidence for the existence of a link between O-GlcNAcylation and NuRD complex formation and function.

In several cervical cancer cell lines, Geo et al. found that OGT knockdown and overexpression altered Snail gene expression [69]. Snail is a DNA-binding transcriptional repressor known to interact with RNAP II and drive epithelial-mesenchymal transition and metastasis when dysregulated [84,85]. Interestingly, the Snail gene was shown to be regulated by the NuRD complex in breast cancer [86]. Thus, OGT, O-GlcNAc cycling, and NuRD may function coordinately to regulate the expression of the Snail gene and potentially other oncogenic and tumor suppressor genes [69,86]. Similar to the OGT containing Sin3A/HDAC complex, regulation may occur at multiple levels. The NuRD complex plays essential roles in chromatin assembly, transcription, cell cycle progression, and genomic stability [87]. The regulation of the NuRD complex by O-GlcNAc cycling represents a largely understudied area of epigenetic gene regulation. Decades of research demonstrate that cancer is not only a gene-based disease but can also arise from epigenetic abnormalities [88]. Aberrant epigenetic gene regulation is a major contributing factor to tumorigenesis, metastasis, and chemotherapy resistance [89]. Thus, understanding O-GlcNAc-mediated NuRD complex formation and function will undoubtedly lead to a better understanding of cancer pathology and potentially other human diseases.

## 5. Defects in O-GlcNAcylation of Transcription Factors Promote Cancer

Almost all RNAP II-associated transcription factors are O-GlcNAcylated, often at multiple sites [53]. O-GlcNAc has been found on RNAP II, RNAP accessory transcription factors, co-activators, co-repressors, and lineage-specific transcription factors. O-GlcNAcylation of these transcriptional proteins affects function in various ways, but overall, the major effects are on their activity, localization, and/or stability. Some transcriptional proteins rely solely on one of these properties, for example, activity. Others use all three mechanisms to regulate their function and influence gene transcription. A growing number of studies highlight the importance of O-GlcNAc in transcription factor function and demonstrate how abnormal O-GlcNAcylation, a common phenotype found in all cancers, leads to abnormal gene expression that favors cancer occurrence, progression, and metastasis. In a non-exhaustive manner, examples of recent studies highlighting the role of O-GlcNAcylation of transcription factors and the co-activator yes-associated protein 1 (YAP) in promoting cancer are discussed below.

### 5.1. O-GlcNAcylation of Transcription Factor Sp1

**Figure 5:**
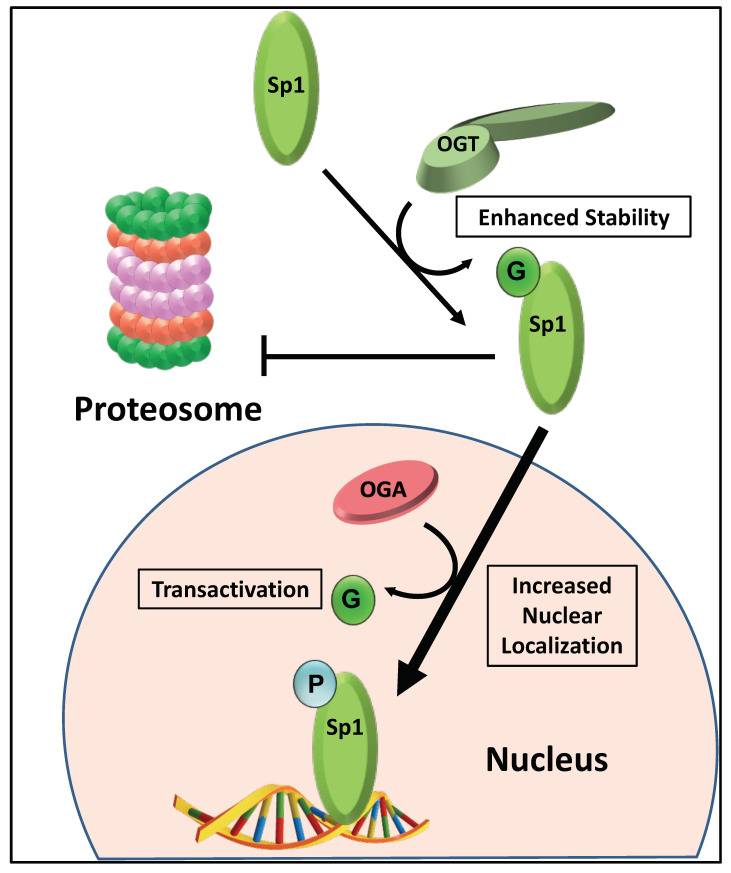
O-GlcNAcylation regulates the stability, nuclear localization, and transactivation of Sp1. Sp1 is a transcription factor that serves as a prototype of how transcription factors are regulated by O-GlcNAcylation at multiple levels. The O-GlcNAcylation of Sp1 (green circle “G”) prevents proteasomal degradation and promotes nuclear localization (large arrow). Once in the nucleus, OGA must remove O-GlcNAc from Sp1 so that it can be phosphorylated to facilitate binding to DNA.

Specificity protein 1 (Sp1) is a transcription factor that is frequently overexpressed in a wide variety of human cancers and contributes to malignant transformation [90]. Sp1 is a ubiquitous and multifunctional transcription factor that targets promoters that lack a TATA box and are GC rich [91]. Sp1 activates the expression of genes that play a role in tumorigenesis via alterations in angiogenesis, cell growth, differentiation, apoptosis, cellular reprogramming, and heat shock protein gene expression following stress [92,93]. Sp1 is a transcription factor that illustrates the role of O-GlcNAc and O-GlcNAc cycling in transcription [94]. For example, O-GlcNAcylation of Sp1 protects the protein from proteasomal degradation and promotes nuclear localization [91]. Once in the nucleus, O-GlcNAc is removed by OGA so that Sp1 can be phosphorylated, which promotes DNA binding [95]. Thus, O-GlcNAcylation of Sp1 appears to play a central role in its nuclear localization, transactivation, and stability (Figure 5).

### 5.2. O-GlcNAcylation of Pluripotent Transcription Factors Sox2 and Oct4

Recent studies have shown that Sox2 and Oct4, two transcription factors critical for the induction and maintenance of pluripotency, are O-GlcNAcylated. In many cancers, Sox2 and Oct4 are inappropriately activated, leading to aberrant expression of downstream target genes, which stimulates tumor growth and tumor recurrence. In mouse embryonic stem cells, Olivier-Van Stichelen et al. found that Oga gene ablation resulted in a significant elevation of Sox2 mRNA transcripts [96]. This suggests that increased cellular O-GlcNAcylation favors the expression of Sox2. Studies of hepatocellular carcinomas support this hypothesis. Cao et al. used RNA-ChIP assays to show that eukaryotic initiation factor 4E (eIF4E), a protein stabilized by O-GlcNAc, was strongly associated with the 5′ G/C rich UTR of the Sox2 transcript, thereby enhancing its translation [97]. At the protein level, Sharma et al. established that O-GlcNAcylation of Sox2 increased its transcriptional activity by enhancing protein stability and nuclear localization [98]. These data suggest that O-GlcNAcylation positively regulates Sox2 at multiple leaves. Oct4 is another key pluripotency transcription factor that often co-regulates genes with Sox2. Constable et al. found that the human Oct4 protein is extensively modified by O-GlcNAc [99]. They identified several novel O-GlcNAc sites that might play a role in controlling Oct4 promoter selectivity. In addition, independent of catalytic function, OGT was suggested to function as a bridge protein between Oct4 and Sox2 to activate transcription at specific promoters. In the hyper-O-GlcNAcylated intracellular environment found in many cancers, these two transcription factors may inappropriately activate pluripotency genes, thereby promoting cancer growth and reoccurrence.

### 5.3. O-GlcNAcylation of Transcription Factors in Breast Cancer

Several studies show that increased O-GlcNAcylation is a key driver of primary and metastatic breast cancer [100,101,102,103,104,105,106]. Approximately 70% of all breast cancers express estrogen receptor (ER) and progesterone receptor (PR). O-GlcNAcylation of ER increases the stability of ER by preventing phosphorylation at O-GlcNAcylated amino acid residues [107,108,109,110]. Interaction between ER and PR enhances ER DNA-binding and target gene expression [111]. Trinca et al. recently found a novel interaction between PR and OGT, leading to O-GlcNAcylation of PR [106]. Elevated levels of O-GlcNAcylation increased PR-mediated transcriptional activity and altered PR-regulated transcriptional networks in breast cancer cells [106]. In addition to ER and PR, Liu et al. reported that the pioneer transcription factor, FOXA1, is modified by O-GlcNAc in several breast cancer cell lines [112]. O-GlcNAcylation of FOXA1 reduces protein stability, leading to the downregulation of the pro-apoptotic Bim protein, thereby inhibiting apoptosis in breast cancer cells [112]. Under the elevated glucose conditions found in breast cancer cells, the expression of key drug resistance proteins increases, all of which are regulated by the Hedgehog pathway. GLI1 and GLI2 are transcription factors that regulate the Hedgehog pathway and were recently found to be modified by OGT [113]. O-GlcNAcylation of these two proteins enhances their transcriptional activity, resulting in activation of the Hedgehog pathway. Collectively, these studies provide explanations as to why elevated O-GlcNAc levels in breast cancer are associated with poor patient prognosis; however, more work is required to probe the intricacies of these gene regulatory mechanisms.

### 5.4. O-GlcNAcylation Regulates the Hippo Pathway Co-Activator YAP

The Hippo pathway is an important signaling pathway that controls organ size by regulating cell proliferation and apoptosis. Dysregulation of this pathway has been linked to tumorigenesis [114,115,116,117]. In mammalian tissues, the major constituents of the Hippo signaling pathway include the transcriptional co-activator yes-associated protein 1 (YAP), nuclear transcription factors (TEAD1/2/3/4), and their upstream kinases (MST1/2 and LATS1/2) [114,118,119]. In response to unfavorable growth conditions, MST1/2 phosphorylates and activates LATS1/2. Activated LATS1/2 then phosphorylates YAP at S127 and/or S381. Phosphorylation of S127 promotes 14-3-3 protein interaction, resulting in cytoplasmic localization of YAP, whereas phosphorylation of S381 leads to YAP degradation by the SCF(β-TrCP) complex [120,121]. Both events inactivate the Hippo pathway [122]. In an activated Hippo pathway, unphosphorylated YAP translocates to the nucleus and functions as a transcriptional co-activator with the TEAD family of transcription factors to stimulate the expression of genes that promote proliferation and impede apoptosis [122,123,124,125].

Recently, Peng et al. demonstrated that this pathway is directly regulated by O-GlcNAcylation [114]. YAP, the core component of the Hippo pathway, interacts with OGT and is O-GlcNAcylated at S109. O-GlcNAcylation of YAP promotes nuclear localization and activation of TEAD downstream target genes. In light of these findings, Zhang et al. reported that O-GlcNAcylation induces transformative phenotypes of liver cancer cells in a YAP-dependent manner [126]. They also identified another O-GlcNAc site on YAP at T241. Mutation of this amino acid residue increased YAP phosphorylation, which led to decreased YAP stability and pro-tumorigenic capacity [126]. Interestingly, both studies found that YAP regulates OGT gene expression and that these two proteins enter into a positive feedback loop to drive YAP O-GlcNAcylation [114]. At least for liver cancer, these studies suggest that elevated cellular O-GlcNAc levels promote YAP activation, and consequently, the expression of pro-tumorigenic genes. Elevated cellular O-GlcNAcylation further exacerbates the YAP-OGT positive feedback loop. Further studies are required to determine whether this positive feedback loop exists in other cancer types.

The transcription factors and co-activator described here are a small cross-section representative of transcription factors and co-activators known the be modified by OGT that function in cancer. More work is required to explore how O-GlcNAcylation of transcription factors affects the expression of genes that regulate cancer occurrence, progression, and metastasis. In the context of nutrient-regulated gene transcription, the data suggest that cells have evolved mechanisms to connect the overall metabolic state of the cell to transcription factor function. Under normal physiologic conditions, this ensures that in times of “feast and famine”, cells elicit the appropriate transcriptional response, and that uncontrolled O-GlcNAcylation of transcription factors plays a role in the onset of cancer.

## 6. Challenges Associated with O-GlcNAc Research

**Figure 6:**
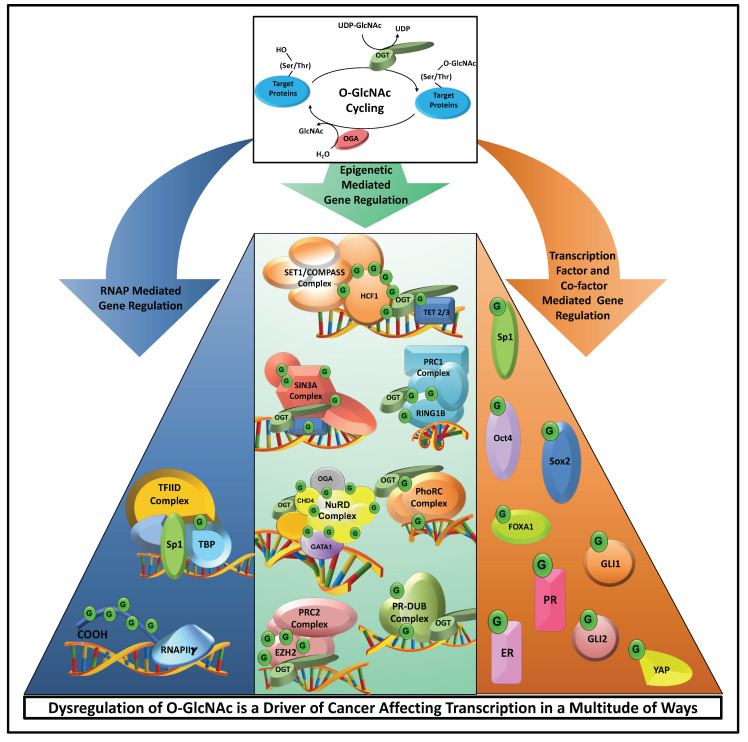
Dysregulation of O-GlcNAc is a driver of cancer by affecting transcription. O-GlcNAcylation is a nutrient-sensitive PTM that modulates gene transcription (green circle “G”). O-GlcNAcylation of the RNAP II CTD affects transcription initiation. It is also found on TBP and numerous RNAP core transcriptional proteins which connects nutrient flux to gene expression. O-GlcNAcylation also influences epigenetic gene regulation via regulation of the SET1/COMPASS, SIN3A, NuRD, PRC1, PRC2, PhoRC, and PR-DUB complexes. Additionally, many transcription factors such as Sp1, Oct4, Sox2, FOXA1, PR, ER, GLI 1, GLI2, and the co-activator YAP all require O-GlcNAcylation to properly regulate their target genes. In general, O-GlcNAcylation of RNAP II, RNAP accessory transcription factors, co-activators, co-repressors, and lineage-specific transcription factors affect their activity, localization, and/or stability. All cancers exhibit aberrant O-GlcNAcylatio; dysregulation of O-GlcNAcylation triggers or exacerbates cancer phenotypes by disrupting normal gene regulation.

O-GlcNAc is a unique driver of cancer since O-GlcNAcylation affects transcription in a multitude of ways on a global level (Figure 6). Under normal physiological conditions, properly controlled dynamic O-GlcNAc cycling serves to connect nutrient availability and metabolic flux to gene expression, allowing cells to respond to environmental demands. The idea of nutrient-regulated gene transcription and its relationship to cancer is an unexplored research area that demands further study to fully understand the underlying causes of cancer. However, several challenges remain. For example, both OGT and OGA are essential for cellular growth, development, and in most cases, survival, making gene knockout studies problematic [127]. Further complicating O-GlcNAc studies is the fact that OGT and OGA levels are transcriptionally linked [128,129]. Experimental manipulations that alter the protein level of one enzyme led to compensatory changes in the protein level of the other enzyme. Thus, attempts to alter the overall cellular level of O-GlcNAcylation are short-lived since cells will adjust the expression of OGT or OGA to compensate and restore O-GlcNAc homeostasis [129,130,131]. Another issue in O-GlcNAc research is the overwhelming number of cellular processes sensitive to O-GlcNAc manipulation. To date, more than 5000 O-GlcNAcylated proteins have been identified. Genetic or pharmacological manipulation of OGT and OGA affect all these proteins making it almost impossible to look at specific O-GlcNAc events. Additionally, because the modification is found on serine and threonine residues, it has extensive crosstalk with phosphorylation. This is problematic, as serine or threonine to alanine substitutions intended to block O-GlcNAcylation also block phosphorylation at these residues. This complicates this experimental approach and can lead to inaccurate data in interpretation.

The technical challenges for studying dynamic O-GlcNAc cycling remain a major obstacle in the field, so there is a need to develop innovative tools for O-GlcNAc studies that reduce the background noise of global O-GlcNAcylation to allow highly specific, focused experiments that yield unbiased data. Newly developed methodology and technical approaches that selectively modify O-GlcNAcylation on a single protein or hone in on the role of O-GlcNAcylation of proteins at specific DNA loci will be indispensable for elucidating the molecular mechanisms that are governed by the addition or removal of this PTM. Recently, Boulard et al. developed a new CRISPR/Cas9 approach that allows for precise targeting of OGA to any genomic sequence of interest [132]. There is no OGT CRISPR/Cas9 tool available to date. These CRISPR/Cas9 approaches have the potential to precisely target the enzymes to specific DNA sequences, thereby avoiding the pleiotropic effects known to complicate conventional gene knockout, knockdown, and over-expression, as well as pharmacological approaches. Based on the sum total of all data generated thus far, it is evident that O-GlcNAc nutrient-regulated gene expression plays an essential role in many transcriptional processes. Thus, research in this area is essential to understand the molecular mechanisms operative to govern transcriptional processes.

## 7. Conclusions

There is a rapidly growing interest in how O-GlcNAcylation contributes to the properties of cancer cells and the progression of cancer. Dynamic O-GlcNAc cycling is commonly elevated in cancer cells. Atypical expression and activities of OGT and OGA have been reported in all human cancers studied thus far [133]. An underlying connection between altered cellular metabolism, a major hallmark of cancer, and dynamic O-GlcNAc cycling has been established. As O-GlcNAc cycling has pleiotropic effects within the cell, OGT and OGA are not good direct targets for therapeutic intervention. However, many of the target proteins and molecular regulatory mechanisms that have been, or will be, discovered may be suitable for therapeutic intervention. Understanding the function of this modification in cancer will be enhanced by the advent of new experimental approaches developed to circumvent the technical hurdles inherent in current O-GlcNAc transcriptional studies.

## References

[1] Hardiville S., Hart G.W. (2014). Nutrient Regulation of Signaling, Transcription, and Cell Physiology by O-GlcNAcylation. Cell Metab..

[2] Brimble S., Wollaston-Hayden E.E., Teo C.F., Morris A.C., Wells L. (2010). The Role of the O-GlcNAc Modification in Regulating Eukaryotic Gene Expression. Curr. Signal. Transduct. Ther..

[3] Torres C.R., Hart G.W. (1984). Topography and Polypeptide Distribution of Terminal N-acetylglucosamine Residues on the Surfaces of Intact Lymphocytes. Evidence for O-linked GlcNAc. J. Biol. Chem..

[4] Hart G.W., Housley M.P., Slawson C. (2007). Cycling of O-linked beta-N-acetylglucosamine on Nucleocytoplasmic Proteins. Nature.

[5] Hart G.W., Slawson C., Ramirez-Correa G., Lagerlof O. (2011). Cross Talk between O-GlcNAcylation and Phosphorylation: Roles in Signaling, Transcription, and Chronic Disease. Annu. Rev. Biochem..

[6] Dong D.L., Hart G.W. (1994). Purification and Characterization of an O-GlcNAc Selective N-acetyl-beta-D-glucosaminidase from Rat Spleen Cytosol. J. Biol. Chem..

[7] Wang T., Birsoy K., Hughes N.W., Krupczak K.M., Post Y., Wei J.J., Lander E.S., Sabatini D.M. (2015). Identification and Characterization of Essential Genes in the Human Genome. Science.

[8] Shafi R., Iyer S.P., Ellies L.G., O’Donnell N., Marek K.W., Chui D., Hart G.W., Marth J.D. (2000). The O-GlcNAc Transferase Gene Resides on the X Chromosome and is Essential for Embryonic Stem Cell Viability and Mouse Ontogeny. Proc. Natl. Acad. Sci. USA.

[9] Keembiyehetty C., Love D.C., Harwood K.R., Gavrilova O., Comly M.E., Hanover J.A. (2015). Conditional Knock-out Reveals a Requirement for O-linked N-Acetylglucosaminase (O-GlcNAcase) in Metabolic Homeostasis. J. Biol. Chem..

[10] Jin L.L., Wybenga-Groot L.E., Tong J., Taylor P., Minden M.D., Trudel S., McGlade C.J., Moran M.F. (2015). Tyrosine Phosphorylation of the Lyn Src Homology 2 (SH2) Domain Modulates Its Binding Affinity and Specificity. Mol. Cell. Proteom. MCP.

[11] Toleman C.A., Schumacher M.A., Yu S.-H., Zeng W., Cox N.J., Smith T.J., Soderblom E.J., Wands A.M., Kohler J.J., Boyce M. (2018). Structural Basis of O-GlcNAc Recognition by Mammalian 14-3-3 Proteins. Proc. Natl. Acad. Sci. USA.

[12] Hanover J.A., Krause M.W., Love D.C. (2010). The Hexosamine Signaling Pathway: O-GlcNAc Cycling in Feast or Famine. Biochim. Biophys. Acta.

[13] Bond M.R., Hanover J.A. (2013). O-GlcNAc Cycling: A Link between Metabolism and Chronic Disease. Annu. Rev. Nutr..

[14] Mondoux M.A., Love D.C., Ghosh S.K., Fukushige T., Bond M., Weerasinghe G.R., Hanover J.A., Krause M.W. (2011). O-linked-N-acetylglucosamine Cycling and Insulin Signaling are Required for the Glucose Stress Response in Caenorhabditis Elegans. Genetics.

[15] Lewis B.A., Burlingame A.L., Myers S.A. (2016). Human RNA Polymerase II Promoter Recruitment in vitro Is Regulated by O-Linked N-Acetylglucosaminyltransferase (OGT). J. Biol. Chem..

[16] Lothrop A.P., Torres M.P., Fuchs S.M. (2013). Deciphering Post-translational Modification Codes. FEBS Lett..

[17] Chiaradonna F., Ricciardiello F., Palorini R. (2018). The Nutrient-Sensing Hexosamine Biosynthetic Pathway as the Hub of Cancer Metabolic Rewiring. Cells.

[18] Haltiwanger R.S., Holt G.D., Hart G.W. (1990). Enzymatic Addition of O-GlcNAc to Nuclear and Cytoplasmic Proteins. Identification of a Uridine Diphospho-N-acetylglucosamine:Peptide Beta-N-acetylglucosaminyltransferase. J. Biol. Chem..

[19] Chaveroux C., Sarcinelli C., Barbet V., Belfeki S., Barthelaix A., Ferraro-Peyret C., Lebecque S., Renno T., Bruhat A., Fafournoux P. (2016). Nutrient Shortage Triggers the Hexosamine Biosynthetic Pathway via the GCN2-ATF4 Signalling Pathway. Sci. Rep..

[20] Wang Z.V., Deng Y., Gao N., Pedrozo Z., Li D.L., Morales C.R., Criollo A., Luo X., Tan W., Jiang N. (2014). Spliced X-box Binding Protein 1 Couples the Unfolded Protein Response to Hexosamine Biosynthetic Pathway. Cell.

[21] Ishino K., Kudo M., Peng W.X., Kure S., Kawahara K., Teduka K., Kawamoto Y., Kitamura T., Fujii T., Yamamoto T. (2018). 2-Deoxy-d-glucose Increases GFAT1 Phosphorylation Resulting in Endoplasmic Reticulum-related Apoptosis via Disruption of Protein N-glycosylation in Pancreatic Cancer Cells. Biochem. Biophys. Res. Commun..

[22] Marshall S., Nadeau O., Yamasaki K. (2004). Dynamic Actions of Glucose and Glucosamine on Hexosamine Biosynthesis in Isolated Adipocytes: Differential Effects on Glucosamine 6-phosphate, UDP-N-acetylglucosamine, and ATP Levels. J. Biol. Chem..

[23] Schleicher E.D., Weigert C. (2000). Role of the Hexosamine Biosynthetic Pathway in Diabetic Nephropathy. Kidney Int. Suppl..

[24] Vasconcelos-Dos-Santos A., Loponte H.F., Mantuano N.R., Oliverira I.A., De Paula I.F., Teixeira L.K., De-Freitas-Junior J.C., Gondim K.C., Heise N., Mohana-Borges R. (2017). Hyperglycemia Exacerbates Colon Cancer Malignancy through Hexosamine Biosynthetic Pathway. Oncogenesis.

[25] Abdel Rahman A.M., Ryczko M., Pawling J., Dennis J.W. (2013). Probing the Hexosamine Biosynthetic Pathway in Human Tumor Cells by Multitargeted Tandem Mass Spectrometry. ACS Chem. Biol..

[26] Akella N.M., Ciraku L., Reginato M.J. (2019). Fueling the Fire: Emerging Role of the Hexosamine Biosynthetic Pathway in Cancer. BMC Biol..

[27] Ma Z., Vosseller K. (2013). O-GlcNAc in Cancer Biology. Amino Acids.

[28] Fardini Y., Dehennaut V., Lefebvre T., Issad T. (2013). O-GlcNAcylation: A New Cancer Hallmark?. Front. Endocrinol..

[29] Slawson C., Hart G.W. (2011). O-GlcNAc Signalling: Implications for Cancer Cell Biology. Nat. Rev. Cancer.

[30] Carter R., Drouin G. (2009). Structural Differentiation of the Three Eukaryotic RNA Polymerases. Genomics.

[31] Koster M.J., Snel B., Timmers H.T. (2015). Genesis of Chromatin and Transcription Dynamics in the Origin of Species. Cell.

[32] Levine M., Cattoglio C., Tjian R. (2014). Looping Back to Leap Forward: Transcription Enters a New Era. Cell.

[33] Lewis B.A., Hanover J.A. (2014). O-GlcNAc and the Epigenetic Regulation of Gene Expression. J. Biol. Chem..

[34] Dahmus M.E. (1996). Reversible Phosphorylation of the C-terminal Domain of RNA Polymerase II. J. Biol. Chem..

[35] Phatnani H.P., Greenleaf A.L. (2006). Phosphorylation and Functions of the RNA Polymerase II CTD. Genes Dev..

[36] Lu H., Flores O., Weinmann R., Reinberg D. (1991). The Nonphosphorylated form of RNA Polymerase II Preferentially Associates with the Preinitiation Complex. Proc. Natl. Acad. Sci. USA.

[37] Kelly W.G., Dahmus M.E., Hart G.W. (1993). RNA Polymerase II is a Glycoprotein. Modification of the COOH-terminal Domain by O-GlcNAc. J. Biol. Chem..

[38] Ranuncolo S.M., Ghosh S., Hanover J.A., Hart G.W., Lewis B.A. (2012). Evidence of the Involvement of O-GlcNAc-modified Human RNA Polymerase II CTD in Transcription in vitro and in vivo. J. Biol. Chem..

[39] Resto M., Kim B.H., Fernandez A.G., Abraham B.J., Zhao K., Lewis B.A. (2016). O-GlcNAcase Is an RNA Polymerase II Elongation Factor Coupled to Pausing Factors SPT5 and TIF1β. J. Biol. Chem..

[40] Love D.C., Ghosh S., Mondoux M.A., Fukushige T., Wang P., Wilson M.A., Iser W.B., Wolkow C.A., Krause M.W., Hanover J.A. (2010). Dynamic O-GlcNAc Cycling at Promoters of *Caenorhabditis elegans* Genes Regulating Longevity, Stress, and Immunity. Proc. Natl. Acad. Sci. USA.

[41] Lewis B.A., Levens D. (2020). O-GlcNAc Transferase Activity is Essential for RNA Pol II Pausing in a Human Cell-Free Transcription System. bioRxiv.

[42] Dynlacht B.D., Hoey T., Tjian R. (1991). Isolation of Coactivators Associated with the TATA-binding Protein That Mediate Transcriptional Activation. Cell.

[43] Hardiville S., Banerjee P.S., Selen Alpergin E.S., Smith D.M., Han G., Ma J., Talbot C.C., Hu P., Wolfgang M.J., Hart G.W. (2020). TATA-Box Binding Protein O-GlcNAcylation at T114 Regulates Formation of the B-TFIID Complex and Is Critical for Metabolic Gene Regulation. Mol. Cell.

[44] Leney A.C., Atmioui D.E., Wu W., Ovaa H., Heck A.J.R. (2017). Elucidating Crosstalk Mechanisms between Phosphorylation and O-GlcNAcylation. Proc. Natl. Acad. Sci. USA.

[45] Wang Z., Gucek M., Hart G.W. (2008). Cross-talk between GlcNAcylation and Phosphorylation: Site-specific Phosphorylation Dynamics in Response to Globally Elevated O-GlcNAc. Proc. Natl. Acad. Sci. USA.

[46] Bourre G., Cantrelle F.X., Kamah A., Chambraud B., Landrieu I., Smet-Nocca C. (2018). Direct Crosstalk between O-GlcNAcylation and Phosphorylation of Tau Protein Investigated by NMR Spectroscopy. Front. Endocrinol..

[47] Morachis J.M., Huang R., Emerson B.M. (2011). Identification of Kinase Inhibitors That Target Transcription Initiation by RNA Polymerase II. Oncotarget.

[48] Segil N., Guermah M., Hoffmann A., Roeder R.G., Heintz N. (1996). Mitotic Regulation of TFIID: Inhibition of Activator-dependent Transcription and Changes in Subcellular Localization. Genes Dev..

[49] Baylin S.B., Jones P.A. (2011). A Decade of Exploring the Cancer Epigenome-Biological and Translational Implications. Nat. Rev. Cancer.

[50] Berger S.L. (2007). The Complex Language of Chromatin Regulation during Transcription. Nature.

[51] Barski A., Cuddapah S., Cui K., Roh T.-Y., Schones D.E., Wang Z., Wei G., Chepelev I., Zhao K. (2007). High-Resolution Profiling of Histone Methylations in the Human Genome. Cell.

[52] Kouzarides T. (2007). Chromatin Modifications and Their Function. Cell.

[53] Hart G.W. (2019). Nutrient Regulation of Signaling and Transcription. J. Biol. Chem..

[54] Olivier-Van Stichelen S., Hanover J.A. (2015). You Are What You Eat: O-linked N-acetylglucosamine in Disease, Development and Epigenetics. Curr. Opin. Clin. Nutr. Metab. Care.

[55] Sakabe K., Wang Z., Hart G.W. (2010). β-N-acetylglucosamine (O-GlcNAc) is Part of the Histone Code. Proc. Natl. Acad. Sci. USA.

[56] Sakabe K., Hart G.W. (2010). O-GlcNAc Transferase Regulates Mitotic Chromatin Dynamics. J. Biol. Chem..

[57] Schübeler D. (2015). Function and Information Content of DNA Methylation. Nature.

[58] Li H.-J., Wang Y., Li B.-X., Yang Y., Guan F., Pang X.-C. (2020). Roles of Ten-eleven Translocation Family Proteins and Their O-linked β-N-acetylglucosaminylated Forms in Cancer Development (Review). Oncol. Lett..

[59] Chen Q., Chen Y., Bian C., Fujiki R., Yu X. (2013). TET2 Promotes Histone O-GlcNAcylation during Gene Transcription. Nature.

[60] Deplus R., Delatte B., Schwinn M.K., Defrance M., Mendez J., Murphy N., Dawson M.A., Volkmar M., Putmans P., Calonne E. (2013). TET2 and TET3 Regulate GlcNAcylation and H3K4 Methylation through OGT and SET1/COMPASS. EMBO J..

[61] Bauer C., Gobel K., Nagaraj N., Colantuoni C., Wang M., Muller U., Kremmer E., Rottach A., Leonhardt H. (2015). Phosphorylation of TET proteins is regulated via O-GlcNAcylation by the O-linked N-acetylglucosamine transferase (OGT). J. Biol. Chem..

[62] Schuettengruber B., Bourbon H.M., Di Croce L., Cavalli G. (2017). Genome Regulation by Polycomb and Trithorax: 70 Years and Counting. Cell.

[63] Varambally S., Dhanasekaran S.M., Zhou M., Barrette T.R., Kumar-Sinha C., Sanda M.G., Ghosh D., Pienta K.J., Sewalt R.G., Otte A.P. (2002). The Polycomb Group Protein EZH2 is Involved in Progression of Prostate Cancer. Nature.

[64] Benard A., Goossens-Beumer I.J., Van Hoesel A.Q., Horati H., Putter H., Zeestraten E.C., Van De Velde C.J., Kuppen P.J. (2014). Prognostic Value of Polycomb Proteins EZH2, BMI1 and SUZ12 and Histone Modification H3K27me3 in Colorectal cancer. PLoS ONE.

[65] Ingham P.W. (1984). A Gene That Regulates the Bithorax Complex Differentially in Larval and Adult Cells of Drosophila. Cell.

[66] Gambetta M.C., Oktaba K., Muller J. (2009). Essential Role of the Glycosyltransferase sxc/Ogt in Polycomb Repression. Science.

[67] Akan I., Love D.C., Harwood K.R., Bond M.R., Hanover J.A. (2016). Drosophila O-GlcNAcase Deletion Globally Perturbs Chromatin O-GlcNAcylation. J. Biol. Chem..

[68] Liu T.-W., Myschyshyn M., Sinclair D.A., Cecioni S., Beja K., Honda B.M., Morin R.D., Vocadlo D.J. (2016). Genome-wide Chemical Mapping of O-GlcNAcylated Proteins in *Drosophila melanogaster*. Nat. Chem. Biol..

[69] Gao J., Yang Y., Qiu R., Zhang K., Teng X., Liu R., Wang Y. (2018). Proteomic analysis of the OGT Interactome: Novel Links to Epithelial-mesenchymal Transition and Metastasis of Cervical Cancer. Carcinogenesis.

[70] Hauri S., Comoglio F., Seimiya M., Gerstung M., Glatter T., Hansen K., Aebersold R., Paro R., Gstaiger M., Beisel C. (2016). A High-Density Map for Navigating the Human Polycomb Complexome. Cell Rep..

[71] Forma E., Jozwiak P., Ciesielski P., Zaczek A., Starska K., Brys M., Krzeslak A. (2018). Impact of OGT Deregulation on EZH2 Target Genes FOXA1 and FOXC1 Expression in Breast Cancer Cells. PLoS ONE.

[72] Jiang M., Xu B., Li X., Shang Y., Chu Y., Wang W., Chen D., Wu N., Hu S., Zhang S. (2019). Correction: O-GlcNAcylation Promotes Colorectal Cancer Metastasis via the miR-101-O-GlcNAc/EZH2 Regulatory Feedback Circuit. Oncogene.

[73] Decourcelle A., Very N., Djouina M., Loison I., Thevenet J., Body-Malapel M., Lelievre E., Coqueret O., Leprince D., El Yazidi-Belkoura I. (2020). O-GlcNAcylation Links Nutrition to the Epigenetic Downregulation of UNC5A during Colon Carcinogenesis. Cancers.

[74] Lo P.-W., Shie J.-J., Chen C.-H., Wu C.-Y., Hsu T.-L., Wong C.-H. (2018). O-GlcNAcylation Regulates the Stability and Enzymatic Activity of the Histone Methyltransferase EZH2. Proc. Natl. Acad. Sci. USA.

[75] Chu C.-S., Lo P.-W., Yeh Y.-H., Hsu P.-H., Peng S.-H., Teng Y.-C., Kang M.-L., Wong C.-H., Juan L.-J. (2014). O-GlcNAcylation Regulates EZH2 Protein Stability and Function. Proc. Natl. Acad. Sci. USA.

[76] Maury J.J., El Farran C.A., Ng D., Loh Y.H., Bi X., Bardor M., Choo A.B. (2015). RING1B O-GlcNAcylation Regulates Gene Targeting of Polycomb Repressive Complex 1 in Human Embryonic Stem Cells. Stem Cell Res..

[77] Zhang Z., Parker M.P., Graw S., Novikova L.V., Fedosyuk H., Fontes J.D., Koestler D.C., Peterson K.R., Slawson C. (2019). O-GlcNAc Homeostasis Contributes to Cell Fate Decisions during Hematopoiesis. J. Biol. Chem..

[78] Yang X., Zhang F., Kudlow J.E. (2002). Recruitment of O-GlcNAc Transferase to Promoters by Corepressor mSin3A: Coupling Protein O-GlcNAcylation to Transcriptional Repression. Cell.

[79] McDonel P., Costello I., Hendrich B. (2009). Keeping Things Quiet: Roles of NuRD and Sin3 Co-repressor Complexes during Mammalian Development. Int. J. Biochem. Cell Biol..

[80] Myers S.A., Panning B., Burlingame A.L. (2011). Polycomb Repressive Complex 2 is Necessary for the Normal Site-specific O-GlcNAc Distribution in Mouse Embryonic Stem Cells. Proc. Natl. Acad. Sci. USA.

[81] Bornelov S., Reynolds N., Xenophontos M., Gharbi S., Johnstone E., Floyd R., Ralser M., Signolet J., Loos R., Dietmann S. (2018). The Nucleosome Remodeling and Deacetylation Complex Modulates Chromatin Structure at Sites of Active Transcription to Fine-Tune Gene Expression. Mol. Cell.

[82] Hoffmann A., Spengler D. (2019). Chromatin Remodeling Complex NuRD in Neurodevelopment and Neurodevelopmental Disorders. Front. Genet..

[83] Zhang Z., Costa F.C., Tan E.P., Bushue N., DiTacchio L., Costello C.E., McComb M.E., Whelan S.A., Peterson K.R., Slawson C. (2016). O-Linked N-Acetylglucosamine (O-GlcNAc) Transferase and O-GlcNAcase Interact with Mi2beta Protein at the Agamma-Globin Promoter. J. Biol. Chem..

[84] Cano A., Pérez-Moreno M.A., Rodrigo I., Locascio A., Blanco M.J., Barrio M.G.d., Portillo F., Nieto M.A. (2000). The Transcription Factor Snail Controls Epithelial–mesenchymal Transitions by Repressing E-cadherin Expression. Nat. Cell Biol..

[85] Batlle E., Sancho E., Franci C., Dominguez D., Monfar M., Baulida J., Garcia De Herreros A. (2000). The Transcription Factor Snail is a Repressor of E-cadherin Gene Expression in Epithelial Tumour Cells. Nat. Cell Biol..

[86] Fujita N., Jaye D.L., Kajita M., Geigerman C., Moreno C.S., Wade P.A. (2003). MTA3, a Mi-2/NuRD complex Subunit, Regulates an Invasive Growth Pathway in Breast Cancer. Cell.

[87] Basta J., Rauchman M. (2015). The Nucleosome Remodeling and Deacetylase Complex in Development and Disease. Transl. Res. J. Lab. Clin. Med..

[88] Goldberg A.D., Allis C.D., Bernstein E. (2007). Epigenetics: A Landscape Takes Shape. Cell.

[89] Sharma S., Kelly T.K., Jones P.A. (2010). Epigenetics in Cancer. Carcinogenesis.

[90] Deniaud E., Baguet J., Mathieu A.L., Pages G., Marvel J., Leverrier Y. (2006). Overexpression of Sp1 Transcription Factor Induces Apoptosis. Oncogene.

[91] Han I., Kudlow J.E. (1997). Reduced O Glycosylation of Sp1 is Associated with Increased Proteasome Susceptibility. Mol. Cell. Biol..

[92] Vellingir B., Iyer M., Devi Subramaniam M., Jayaramayya K., Siama Z., Giridharan B., Narayanasamy A., Abdal Dayem A., Cho S.G. (2020). Understanding the Role of the Transcription Factor Sp1 in Ovarian Cancer: From Theory to Practice. Int. J. Mol. Sci..

[93] Banerjee S., Sangwan V., McGinn O., Chugh R., Dudeja V., Vickers S.M., Saluja A.K. (2013). Triptolide-induced Cell Death in Pancreatic Cancer is Mediated by O-GlcNAc Modification of Transcription Factor Sp1. J. Biol. Chem..

[94] Yang X., Qian K. (2017). Protein O-GlcNAcylation: Emerging Mechanisms and Functions. Nat. Rev. Mol. Cell Biol..

[95] Yang X., Su K., Roos M.D., Chang Q., Paterson A.J., Kudlow J.E. (2001). O-linkage of N-acetylglucosamine to Sp1 Activation Domain Inhibits Its Transcriptional Capability. Proc. Natl. Acad. Sci. USA.

[96] Olivier-Van Stichelen S., Wang P., Comly M., Love D.C., Hanover J.A. (2017). Nutrient-driven O-linked N-acetylglucosamine (O-GlcNAc) Cycling Impacts Neurodevelopmental Timing and Metabolism. J. Biol. Chem..

[97] Cao B., Duan M., Xing Y., Liu C., Yang F., Li Y., Yang T., Wei Y., Gao Q., Jiang J. (2019). O-GlcNAc Transferase Activates Stem-like Cell Potential in Hepatocarcinoma through O-GlcNAcylation of Eukaryotic Initiation Factor 4E. J. Cell. Mol. Med..

[98] Sharma N.S., Gupta V.K., Dauer P., Kesh K., Hadad R., Giri B., Chandra A., Dudeja V., Slawson C., Banerjee S. (2019). O-GlcNAc Modification of Sox2 Regulates Self-renewal in Pancreatic Cancer by Promoting Its Stability. Theranostics.

[99] Constable S., Lim J.M., Vaidyanathan K., Wells L. (2017). O-GlcNAc Transferase Regulates Transcriptional Activity of Human Oct4. Glycobiology.

[100] Gu Y., Mi W., Ge Y., Liu H., Fan Q., Han C., Yang J., Han F., Lu X., Yu W. (2010). GlcNAcylation Plays an Essential Role in Breast Cancer Metastasis. Cancer Res..

[101] Champattanachai V., Netsirisawan P., Chaiyawat P., Phueaouan T., Charownwattanasatien R., Chokchaichamnankit D., Punyarit P., Srisomsap C., Svasti J. (2013). Proteomic Analysis and Abrogated Expression of O-GlcNAcylated Proteins Associated with Primary Breast Cancer. Proteomics.

[102] Ferrer C.M., Lynch T.P., Sodi V.L., Falcone J.N., Schwab L.P., Peacock D.L., Vocadlo D.J., Seagroves T.N., Reginato M.J. (2014). O-GlcNAcylation Regulates Cancer Metabolism and Survival Stress Signaling via Regulation of the HIF-1 Pathway. Mol. Cell.

[103] Krzeslak A., Forma E., Bernaciak M., Romanowicz H., Brys M. (2012). Gene Expression of O-GlcNAc Cycling Enzymes in Human Breast Cancers. Clin. Exp. Med..

[104] Caldwell S.A., Jackson S.R., Shahriari K.S., Lynch T.P., Sethi G., Walker S., Vosseller K., Reginato M.J. (2010). Nutrient Sensor O-GlcNAc Transferase Regulates Breast Cancer Tumorigenesis through Targeting of the Oncogenic Transcription Factor FoxM1. Oncogene.

[105] Sodi V.L., Khaku S., Krutilina R., Schwab L.P., Vocadlo D.J., Seagroves T.N., Reginato M.J. (2015). mTOR/MYC Axis Regulates O-GlcNAc Transferase Expression and O-GlcNAcylation in Breast Cancer. Mol. Cancer Res..

[106] Trinca G.M., Goodman M.L., Papachristou E.K., D’Santos C.S., Chalise P., Madan R., Slawson C., Hagan C.R. (2018). O-GlcNAc-Dependent Regulation of Progesterone Receptor Function in Breast Cancer. Horm. Cancer.

[107] Jiang M.S., Hart G.W. (1997). A Subpopulation of Estrogen Receptors are Modified by O-linked N-acetylglucosamine. J. Biol. Chem..

[108] Cheng X., Hart G.W. (2000). Glycosylation of the Murine Estrogen Receptor-alpha. J. Steroid Biochem. Mol. Biol..

[109] Cheng X., Hart G.W. (2001). Alternative O-glycosylation/O-phosphorylation of Serine-16 in Murine Estrogen Receptor Beta: Post-translational Regulation of Turnover and Transactivation Activity. J. Biol. Chem..

[110] Ozcan S., Andrali S.S., Cantrell J.E. (2010). Modulation of Transcription Factor Function by O-GlcNAc Modification. Biochim. Biophys. Acta.

[111] Carroll J.S., Hickey T.E., Tarulli G.A., Williams M., Tilley W.D. (2016). Deciphering the Divergent Roles of Progestogens in Breast Cancer. Nat. Rev. Cancer.

[112] Liu Y., Wang X., Zhu T., Zhang N., Wang L., Huang T., Cao Y., Li W., Zhang J. (2019). Resistance to Bortezomib in Breast Cancer Cells That Downregulate Bim through FOXA1 O-GlcNAcylation. J. Cell. Physiol..

[113] Das S., Bailey S.K., Metge B.J., Hanna A., Hinshaw D.C., Mota M., Forero-Torres A., Chatham J.C., Samant R.S., Shevde L.A. (2019). O-GlcNAcylation of GLI Transcription Factors in Hyperglycemic Conditions Augments Hedgehog Activity. Lab. Invest..

[114] Peng C., Zhu Y., Zhang W., Liao Q., Chen Y., Zhao X., Guo Q., Shen P., Zhen B., Qian X. (2017). Regulation of the Hippo-YAP Pathway by Glucose Sensor O-GlcNAcylation. Mol. Cell.

[115] Halder G., Johnson R.L. (2011). Hippo Signaling: Growth Control and Beyond. Development.

[116] Pan D. (2010). The Hippo Signaling Pathway in Development and Cancer. Dev. Cell.

[117] Zhao B., Li L., Lei Q., Guan K.L. (2010). The Hippo-YAP Pathway in Organ Size Control and Tumorigenesis: An Updated Version. Genes Dev..

[118] Yu F.X., Zhao B., Guan K.L. (2015). Hippo Pathway in Organ Size Control, Tissue Homeostasis, and Cancer. Cell.

[119] Zhao B., Tumaneng K., Guan K.-L. (2011). The Hippo Pathway in Organ Size Control, Tissue Regeneration and Stem Cell Self-renewal. Nat. Cell Biol..

[120] Zhao B., Wei X., Li W., Udan R.S., Yang Q., Kin J., Xie J., Ikenoue T., Yu J., Li L. (2007). Inactivation of YAP Oncoprotein by the Hippo Pathway is Involved in Cell Contact Inhibition and Tissue Growth Control. Genes Dev..

[121] Zhoa B., Li L., Tumaneng K., Wang C.Y., Guan K.L. (2010). A Coordinated Phosphorylation by Lats and CK1 Regulates YAP Stability through SCF(beta-TRCP). Genes Dev..

[122] Meng Z., Moroishi T., Guan K.L. (2016). Mechanisms of Hippo Pathway Regulation. Genes Dev..

[123] Lamar J.M., Stern P., Liu H., Schindler J.W., Jiang Z.-G., Hynes R.O. (2012). The Hippo Pathway Target, YAP, Promotes Metastasis through Its TEAD-interaction Domain. Proc. Natl. Acad. Sci. USA.

[124] Gumbiner B.M., Kim N.-G. (2014). The Hippo-YAP Signaling Pathway and Contact Inhibition of Growth. J. Cell Sci..

[125] Zanconato F., Cordenonsi M., Piccolo S. (2016). YAP/TAZ at the Roots of Cancer. Cancer Cell.

[126] Zhang X., Qiao Y., Wu Q., Chen Y., Zou S., Liu X., Zhu G., Zhao Y., Chen Y., Yu Y. (2017). The Essential Role of YAP O-GlcNAcylation in High-glucose-stimulated Liver Tumorigenesis. Nat. Commun..

[127] O’Donnell N., Zachara N.E., Hart G.W., Marth J.D. (2004). Ogt-dependent X-chromosome-linked Protein Glycosylation is a Requisite Modification in Somatic Cell Function and Embryo Viability. Mol. Cell. Biol..

[128] Slawson C., Copeland R.J., Hart G.W. (2010). O-GlcNAc Signaling: A Metabolic Link between Diabetes and Cancer?. Trends Biochem. Sci..

[129] Zhang Z., Tan E.P., VandenHull N.J., Peterson K.R., Slawson C. (2014). O-GlcNAcase Expression is Sensitive to Changes in O-GlcNAc Homeostasis. Front. Endocrinol..

[130] Kazemi Z., Chang H., Haserodt S., McKen C., Zachara N.E. (2010). O-linked Beta-N-acetylglucosamine (O-GlcNAc) Regulates Stress-induced Heat Shock Protein Expression in a GSK-3beta-Dependent Manner. J. Biol. Chem..

[131] Slawson C., Zachara N.E., Vosseller K., Cheung W.D., Lane M.D., Hart G.W. (2005). Perturbations in O-linked Beta-N-acetylglucosamine Protein Modification Cause Severe Defects in Mitotic Progression and Cytokinesis. J. Biol. Chem..

[132] Boulard M., Rucli S., Edwards J.R., Bestor T.H. (2020). Methylation-directed Glycosylation of Chromatin Factors Represses Retrotransposon Promoters. Proc. Natl. Acad. Sci. USA.

[133] Forma E., Jóźwiak P., Bryś M., Krześlak A. (2014). The Potential Role of O-GlcNAc Modification in Cancer Epigenetics. Cell. Mol. Biol. Lett..

